# Extracellular vesicles derived from stressed beta cells mediate monocyte activation and contribute to islet inflammation

**DOI:** 10.3389/fimmu.2024.1393248

**Published:** 2024-07-24

**Authors:** Mette C. Dekkers, Joost M. Lambooij, Xudong Pu, Raphael R. Fagundes, Agustin Enciso-Martinez, Kim Kats, Ben N. G. Giepmans, Bruno Guigas, Arnaud Zaldumbide

**Affiliations:** ^1^ Department of Cell and Chemical Biology, Leiden University Medical Center, Leiden, Netherlands; ^2^ Leiden University Center of Infectious Diseases, Leiden University Medical Center, Leiden, Netherlands; ^3^ Oncode institute, Leiden University Medical Center, Leiden, Netherlands; ^4^ Amsterdam Vesicle Center, Biomedical Engineering and Physics and Laboratory of Experimental Clinical Chemistry, Amsterdam University Medical Center (UMC), University of Amsterdam, Amsterdam, Netherlands; ^5^ Department of Biomedical Sciences, University Medical Center Groningen, University of Groningen, Groningen, Netherlands

**Keywords:** type 1 diabetes, er stress, extracellular vesicles, monocytes, inflammation

## Abstract

**Objective:**

Beta cell destruction in type 1 diabetes (T1D) results from the combined effect of inflammation and recurrent autoimmunity. In recent years, the role played by beta cells in the development of T1D has evolved from passive victims of the immune system to active contributors in their own destruction. We and others have demonstrated that perturbations in the islet microenvironment promote endoplasmic reticulum (ER) stress in beta cells, leading to enhanced immunogenicity. Among the underlying mechanisms, secretion of extracellular vesicles (EVs) by beta cells has been suggested to mediate the crosstalk with the immune cell compartment.

**Methods:**

To study the role of cellular stress in the early events of T1D development, we generated a novel cellular model for constitutive ER stress by modulating the expression of *HSPA5*, which encodes BiP/GRP78, in EndoC-βH1 cells. To investigate the role of EVs in the interaction between beta cells and the immune system, we characterized the EV miRNA cargo and evaluated their effect on innate immune cells.

**Results:**

Analysis of the transcriptome showed that *HSPA5* knockdown resulted in the upregulation of signaling pathways involved in the unfolded protein response (UPR) and changes the miRNA content of EVs, including reduced levels of miRNAs involved in IL-1β signaling. Treatment of primary human monocytes with EVs from stressed beta cells resulted in increased surface expression of CD11b, HLA-DR, CD40 and CD86 and upregulation of IL-1β and IL-6.

**Conclusion:**

These findings indicate that the content of EVs derived from stressed beta cells can be a mediator of islet inflammation.

## Introduction

Type 1 diabetes (T1D) is a chronic disease characterized by the selective destruction of beta cells located in the pancreatic islets of Langerhans. Although T1D is classically described as an autoimmune disease in which autoreactive T cells escape thymic negative selection, recent evidence implicates beta cells themselves as drivers of the disease (1). Increased expression of CHOP, a marker for endoplasmic reticulum (ER) stress, in beta cells before immune cell infiltration suggests that beta cell stress is present at early stages of T1D development (2). Disruption of ER homeostasis caused by environmental factors can lead to the induction of ER stress, and subsequently activation of the unfolded protein response (UPR) via the release of chaperone BiP from the ER transmembrane sensors (i.e. inositol-requiring protein 1α (IRE1α), protein kinase RNA-like endoplasmic reticulum kinase (PERK), and activating transcription factor 6 (ATF6)) (3). Induction of the UPR aims at lowering pressure on the ER by reducing translation and promoting protein degradation through the ER-associated degradation (ERAD) pathway. However, several studies have demonstrated that this adaptive phase to stress can lead to the generation of neoantigens which can be recognized by the immune surveillance machinery (4–7).

Increasing evidence indicates an important role for extracellular vesicles (EVs) in the crosstalk between cells in the pancreatic islet (8–11). EVs are lipid membrane vesicles secreted by the cell and consist of exosomes, microvesicles and apoptotic bodies, which are categorized based on size, biogenesis and release pathway (12). These vesicles contain a broad repertoire of biologically active cargo molecules, including DNA, RNA, lipids and proteins, serving as a means to convey information to recipient cells (13). The uptake of EVs by surrounding cells is mediated by endocytosis, fusion with the plasma membrane or binding to surface receptors. EVs can mediate local as well as systemic effects, as they are rapidly transported in interstitial fluids and have been detected in draining lymph nodes within minutes after secretion (14, 15). Interestingly, previous studies have shown that ER stress may result in increased formation of multivesicular bodies and release of EVs (16, 17). The enhanced secretion might complement the function of ERAD and autophagy to clear misfolded proteins from stressed cells, and to restore homeostasis (18). However, increased EV secretion might inadvertently enhance beta cell visibility to patrolling immune cells in case of cellular damage (8, 11). Moreover, beta cell EVs have been found in blood plasma, which suggests that they can also reach circulating immune cells (19).

In the present study, we investigate the EV-mediated crosstalk between stressed beta cells and monocytes. Our data shows that EVs produced by EndoC-βH1 cells under ER stress contain an altered miRNA profile and are able to promote the activation of primary human monocytes. These findings support the concept that stressed7nbsp;beta cells can induce activation of innate immune cells through the release of EVs, which may contribute to the generation of a proinflammatory microenvironment and initiation of autoimmunity in the development of T1D.

## Materials and methods

### Cell culture

EndoC-βH1 cells, obtained from Dr. Raphael Scharfmann (Paris Descartes University, France) (20), were maintained in low glucose DMEM (Gibco, 31885023), serum-free, supplemented with 5.5 μg/ml human transferrin (Sigma, T8158), 10 mM nicotinamide, 6.7 ng/ml selenite (Sigma, S5261), 50 μM β-mercaptoethanol, 2% human albumin (Alburex 20), 100 units/ml penicillin and 100 μg/ml streptomycin. Cells were seeded in extracellular matrix (Sigma, E1270) and fibronectin (Sigma, F1141) coated culture plates.

### Lentiviral transduction with shRNA

To induce ER stress, EndoC-βH1 cells were transduced with short hairpin RNA against *HSPA5* (Sigma, TRCN0000218646) or non-target control short hairpin RNA (Sigma, SHC002) obtained from the Mission shRNA library, at MOI=1 using polybrene (8 ug/ml). Transduced cells were selected using puromycin (3 ug/ml) from day 3-6 post-transduction. On day 6, the cells were replated and cultured in puromycin-free medium for 2 days before further use.

### RNA isolation and qPCR

RNA was isolated using NucleoSpin RNA kit (Bioké) according to the manufacturer’s protocol. After RNA isolation, cDNA was synthesized using Superscript II Reverse Transcriptase (Invitrogen). Targeted gene expression levels were determined by qPCR using iQ SYBR^®^ Green Supermix (Bio-Rad) and gene-specific primers (**Table 1**). qPCRs were run on a CFX Connect Real-Time PCR Detection System (Bio-Rad). Gene expression levels were normalized to the level of housekeeping genes beta-actin (monocytes) or GAPDH (EndoC-βH1 cells) and presented as 2^-ΔΔCT^ to the respective controls.

**Table 1:** Primers used for qPCR.

Gene	Forward primer (5’-3’)	Reverse primer (5’-3’)
HSPA5	GAC GCT GGA ACT ATT GCT GG	CTC CCT CTT ATC CAG GCC AT
XBP1-spliced	CTG AGT CCG CAG CAG GTG	GAG ATG TTC TGG AGG GGT GA
DDIT3	GAC CTG CAA GAG GTC CTG TC	CTC CTC CTC AGT CAG CCA AG
GAPDH	ACA GTC AGC CGC ATC TTC TT	AAT GAA GGG GTC ATT GAT GG
IL1B	AAC ACG CAG GAC AGG TAC AG	GAG CAA CAA GTG GTG TTC TTC
IL6	TTG TCA TGT CCT GCA GCC	GAG TAG TGA GGA ACA AGC CAG
TNF	GAG GGT TTG CTA CAA CAT GGG	TCC CCA GGG ACC TCT CTC TA
ACTB	GCT GTG CTA CGT CGC CCT GG	ACA GGA CTC CAT GCC CAG GAA GG

### Cell viability EndoC-βH1

Viability of EndoC-βH1 cells was assessed using PE Annexin V Apoptosis Detection Kit with 7-AAD (BioLegend), according to manufacturer’s instructions. Cells were analyzed on a BD^®^ LSR-II Flow Cytometer (BD Biosciences).

### Glucose-stimulated insulin secretion assay

EndoC-βH1 cells were seeded at 300.000 cells per well in a 12-wells plate. Cells were starved overnight in ULTI-ST^®^ medium (Human Cell Design). Next, cells were washed with βKREBS^®^-BSA solution (Human Cell Design) and incubated in βKREBS^®^-BSA for 60 minutes at 37°C + 5% CO_2_. Supernatant was collected and the cells were incubated in βKREBS^®^-BSA with 20 mM glucose for an additional 60 minutes at 37°C + 5% CO_2_. Subsequently, supernatant was collected for high glucose conditions and cells were lysed in 0.5 M EDTA, 50 mM HEPES, 1% Triton X-100.

### Insulin ELISA

Insulin secretion in the supernatant of EndoC-βH1 cells was quantified using a human insulin ELISA kit (Mercodia) according to manufacturer’s instructions.

### RNA sequencing

For unbiased transcriptomic analysis, RNA from EndoC-βH1 shCTRL and shHSPA5 (n=3) was isolated using Nucleospin RNA Kit (Bioke) according to the manufacturer’s guidelines. Quality control, library preparation and sequencing were performed by Biomarker Technologies (BMK) GmbH. In brief, purity, concentration and integrity of RNA samples were examined by NanoDrop (Thermo Fisher Scientific), Qubit 2.0 (Life Technologies) and Agilent 2100 Bioanalyzer (Agilent). Sequencing libraries were generated using NEBNext Ultra RNA Library Prep Kit for Illumina (NEB) following manufacturer’s recommendations and index codes were added to attribute sequences to each sample. Clustering of the index-coded samples was performed on a cBot Cluster Generation System using TruSeq PE Cluster Kit v4-cBot-HS (Illumina). After cluster generation, the library preparations were sequenced on an Illumina NovaSeq 6000 system (Illumina) with a sequencing depth of 20M reads per sample.

### RNAseq transcriptome analysis

Clean reads were obtained by trimming adapters and removing nucleotides with low quality. HISAT2 (21) was used for alignment on the reference genome (Homo_sapiens.GCF_000001405.40_GRCh38.p14). Gene expression levels were quantified as fragments per kilobase of transcript per million fragments mapped (FPKM). Following analyses were conducted using R version 3.1.3 (22). Correlation between biological replicates was assessed by principal component analysis (PCA), using the R package *factoextra* (23). Differential expression analysis was performed using the R package *DESeq2* (24) with a threshold of |log2(FC)|≥1.0;FDR ≤ 0.01. Heatmaps were generated using *pheatmap* (25) and volcano plots were created using GraphPad Prism version 10.1.1. To identify affected biological pathways, the differentially expressed genes were subjected to gene ontology enrichment analysis using the Gene Ontology knowledgebases Gene Ontology Biological Process (GO-BP), Gene Ontology Cellular Component (GO-CC), Gene Ontology Molecular Function (GO-MF) (26, 27) and Reactome (28), implemented by *clusterProfiler* package (29) and *ReactomePA* (30). Figures of gene ontology analysis results were generated using *enrichplot* (31).

### Extracellular vesicle isolation

After 48 hours of cell culture, to allow for secretion of EVs, conditioned serum-free cell culture medium was collected and centrifuged at 500g for 10 minutes to remove cell debris. The supernatant was concentrated using Amicon™ Ultra-15 Centrifugal Filter Units (Merck Millipore) with sequential centrifugations of 11 minutes at 3,200 g, until the concentrate reached a volume of 500 µl. EVs were isolated from the concentrate by size exclusion chromatography (SEC) using qEVoriginal/35nm Legacy columns (Izon). After 2.5 mL of buffer collection (flow-through fraction), 2.4 mL was collected for EVs (EV fraction). The protein concentration of lysed EVs was determined by Pierce™ BCA Protein Assay Kit (Thermo Fisher Scientific). EVs were stored in filtered PBS at -80°C in Protein LoBind tubes (Eppendorf) to reduce attachment of EVs to the plastic.

### Transmission electron microscopy

Negative stain and image acquisition of EV suspensions were performed as described previously (32). A droplet of 10 µl EV suspension was transferred to a 150 mesh Formvar coated copper grid (0150-Cu, Electron Microscopy Sciences) and incubated for 20 minutes, then drained with filter paper and stained with ammonium molybdate for 10 minutes. After draining, the grids were washed on a droplet of double distilled water for 10 seconds. Samples were imaged on a Talos F200i (Thermo Fisher Scientific).

### Nanoparticle tracking analysis

Particle size distribution and concentration were assessed by nanoparticle tracking analysis (NTA) using a NanoSight NS300 instrument (Malvern Panalytical Ltd.) equipped with a 488 nm blue laser and a sCMOS camera. NanoSight software (NTA 3.2 Dev Build 3.2.16) was used for recording and analysis. In brief, the EV and protein fractions were diluted with filtered PBS in accordance to the detection range (20–100 particles/frame). Three replicates of each fraction were loaded and three 60-second videos were captured for each replicate with the following settings: syringe flow rate 100, camera level 10, temperature 22°C, and viscosity 1.0 cP (water). The following settings were employed for analysis: screen gain 10 and detection threshold 12.

### Protein precipitation of extracellular vesicles by trichloroacetic acid

To precipitate EV proteins, EVs were incubated in a solution of 2 mg/mL sodium deoxycholate, followed by supplementation with cold 100% (w/v) trichloroacetic acid (TCA) to a final concentration of 20%. After incubation for 30 minutes at 4°C, samples were centrifugated at 16,000g for 10 minutes at 4°C and supernatant was removed. Protein pellets were washed twice with 1 mL of 100% ice cold acetone and then dried at room temperature for 10 minutes. Pellets were resuspended in loading buffer (bromophenol blue 0.05%, glycerol 10%, SDS 2%, Tris-HCl 0.05M pH 6.8) and heated for 5 minutes at 95°C.

### Western blot

Proteins extracts (30µg) were separated by 10% sodium dodecyl sulfate–polyacrylamide gel electrophoresis and subsequently transferred onto nitrocellulose membranes (1704158, Bio-Rad). After blocking with 5% milk in Tris-buffered saline supplemented with Tween-20 (TBST) at room temperature for 1 hour, the membranes were incubated overnight at 4°C with mouse anti-human CD63 antibody (sc-5275, Santa Cruz Biotechnology), CD81 antibody (349502, BioLegend) or Calnexin antibody (sc-23954, Santa Cruz Biotechnology) at a dilution of 1:1000 in 5% milk. After washing with TBST, the membranes were incubated with horseradish peroxidase (HRP)-conjugated goat anti-mouse IgG secondary antibody (31430, Thermo Fisher Scientific) at a dilution of 1:5000, followed by washing with TBST. An enhanced chemiluminescence (ECL) substrate (34075, Thermo Fisher Scientific) was used for imaging.

### Small RNA sequencing

Total RNA was extracted from EVs derived from EndoC-βH1 shCTRL and shHSPA5 (n=3) using Nucleospin miRNA kit (Bioké). Purity, concentration and integrity of RNA samples were examined by NanoDrop (Thermo Fisher Scientific), Qubit 2.0 (Life Technologies) and Agilent 2100 Bioanalyzer (Agilent). The library was constructed using an NEB Next Small RNA Sample Library Prep Kit (NEB), index codes were added to attribute sequences to each sample, and then sequenced on an Illumina NovaSeq 6000 platform (Illumina) with a sequencing depth of 20M reads per sample. Library preparation and sequencing was performed by Biomarker Technologies (BMK) GmbH.

### miRNAseq analysis

Adapter sequences, low-quality sequences and reads with lengths smaller than 18 nt or longer than 30 nt were removed to extract clean data. Clean reads were mapped to Silva, GtRNAdb, Rfam and Repbase to remove ncRNAs, including rRNA, tRNA, snRNA, snoRNA and repeated sequences. The remaining unannotated reads were regarded as reads containing miRNAs. Unannotated reads were mapped to reference genome Homo_sapiens.GRCh38_release95 with Bowtie (33) to obtain their positions. Known miRNAs were identified by comparing mapped reads with mature miRNA in miRBase (v22) database. Mature miRNA sequences with 2 nt up-stream and 5 nt down-stream were used in searching. Mapped reads with maximum 1 mis-match were regarded as matching to known miRNA. The remaining reads were analyzed by miRDeep2 (34) to predict novel miRNAs based on specific species. The gene targets of the identified miRNAs were predicted based on miRWalk (35), filtering for a binding probability of at least 0.95, 3’UTR binding sites and validation in the miRDB database. Differential expressed miRNAs were determined using the *DESeq2* package with a threshold of FDR ≤ 0.05. PCA, generation of volcano plots, heatmaps, Gene Ontology and Reactome enrichment analysis were performed in R, as described above. Results from Gene Ontology and Reactome were scanned for the following terms: “interleukin”, “insulin”, “Insulin” and “IRS”. Dot plots were generated from these selected ontologies using the *ggplot2* package (36).

### Monocyte isolation and stimulation

Human monocytes were isolated from concentrated peripheral blood (buffy coat) donated by healthy anonymous volunteers at Sanquin bloodbank (Amsterdam, Netherlands) as described previously (37, 38). Briefly, peripheral blood mononuclear cells (PBMCs) were isolated from blood diluted in equal volume of HBSS by means of a Ficoll-Paque density gradient. Monocytes were subsequently isolated from PBMCs by positive selection using magnetic-activated cell sorting (MACS) with CD14-beads (Miltenyi Biotec). Isolated monocytes were treated with 100 ug/mL of EVs (isolated as described under “2.6 Extracellular vesicle isolation”, corresponding to 5.81E+08/ml for shCTRL EVs, and 5.76E+08/ml for shHSPA5 EVs (for monocytes treated with EV batch 2) as defined by NTA) or equal volume of PBS (no EV control) in RPMI 1640 (Invitrogen) supplemented with 5% FBS (Serana), 2mM L-glutamine (Sigma), 100U/mL penicillin (Eureco-Pharma) and 100ug/mL streptomycin (Sigma) for 24 hours at 37°C + 5% CO_2_. After incubation, the monocytes were washed with PBS and subsequently mounted for confocal imaging, stained for analysis by flow cytometry or lysed for mRNA isolation.

### Extracellular vesicle uptake

To examine EV uptake by monocytes, EVs or PBS (no EV control) were stained in Vybrant™ DiI staining solution (1:200, Invitrogen) and incubated for 20 minutes at 37°C. Unbound dye was removed using EV-Spinner (HansaBioMed Life Sciences). After 18h of incubation with 100 ug/ml EVs or equal volume of control at 37°C + 5% CO_2_, monocytes were washed with PBS and fixed using 2% paraformaldehyde (PFA) for 15 minutes at room temperature. To assess EV uptake by imaging, cells were washed with PBS, mounted in ProLong™ Gold Antifade Mountant with DNA Stain DAPI (Invitrogen) and imaged on a Zeiss LSM900 confocal microscope with Airyscan (Zeiss) using 405 nm and 561 nm excitation lasers. To quantify EV uptake by flow cytometry, cells were washed with PBS, resuspended in PBS supplemented with 0.5% bovine serum albumin (BSA, Roche), 2mM ethylenediamine tetraacetic acid (EDTA, Sigma) (PBS/BSA/EDTA) and analyzed on a BD^®^ LSR-II Flow Cytometer (BD Biosciences).

### Flow cytometry monocytes

To assess cell surface expression of activation markers, stimulated monocytes were harvested and processed for flow cytometry as follows. Samples were washed twice with PBS by pelleting the cells at 450g for 5 minutes at 4°C, discarding the supernatant and resuspending in PBS. After washing, the samples were stained using a Zombie NIR Fixable viability kit (Biolegend) in PBS supplemented with monocyte blocker (Biolegend) for 20 minutes at room temperature after which samples were washed with PBS and fixed using 2% PFA in PBS for 15 minutes at room temperature. Next, the samples were washed and incubated with a cocktail of antibodies directed against CD11b, HLA-DR, CD40, CD80 and CD86 in PBS/BSA/EDTA and brilliant stain buffer plus (BD Biosciences) for 30 minutes at 4°C. Finally, the samples were washed using PBS, resuspended in PBS/BSA/EDTA and acquired on a Cytek 5-laser Aurora spectral flow cytometer (Cytek Biosciences). For a full list of antibodies and reagents used for flow cytometry, please refer to **Table 2**.

**Table 2:** List of reagents used for flow cytometry.

Target	Fluorochrome	Clone	Supplier	Catalog. Number	Dilution
CD11b	BV570	ICRF44	Biolegend	301325	1:500
CD40	BV650	5C3	Biolegend	334338	1:500
CD80	PE-Cy5	L307.4	BD Biosciences	559370	1:100
CD86	FITC	2331 (FUN-1)	BD Biosciences	560958	1:500
HLA-DR	BV605	G46-6	BD Biosciences	560958	1:500
Viability	Zombie-NIR	N/A	Biolegend	423106	1:1000

### Data analysis

Spectral unmixing of flow cytometry data was performed using SpectroFlo software v3.2.1 (Cytek Biosciences). Data processing and analysis of all flow cytometry data were performed using FlowJo v10.1.0 (BD Biosciences). T tests and ANOVA were performed using Graphpad Prism version 9.3.1, unless otherwise indicated.

A p value of ≤0.05 was considered statistically significant and is indicated by *. P value of ≤0.01 is indicated by **, p value of ≤0.001 is indicated by *** and a p value of ≤0.0001 is indicated by ****.

## Results

### Knockdown of *HSPA5* results in activation of the ER stress response

**Figure 1:**
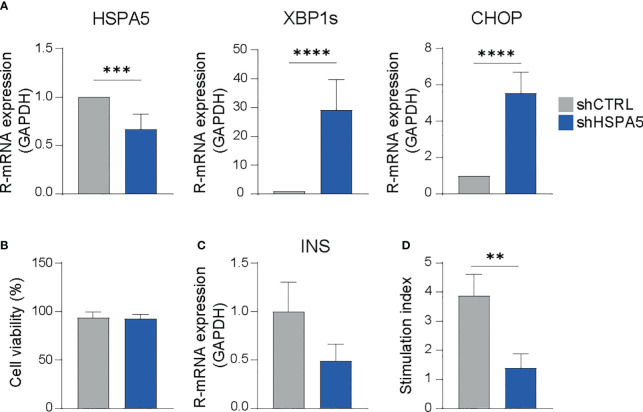
*HSPA5* knockdown in EndoC-βH1 cells results in activation of the ER stress response. **(A)** Validation of *HSPA5* knockdown and upregulation of ER stress markers *XBP1s* and *CHOP* as evaluated by qPCR. Bars represent the mean with standard deviation (n=7). Statistical significance was tested using unpaired T-tests. **(B)** Viability of shHSPA5 and shCTRL cells, as determined by staining for Annexin V and 7AAD and measured by flow cytometry. Bars represent the mean with standard deviation (n=3). Statistical significance was tested using unpaired T-test. **(C)** Gene expression of *INS* in shHSPA5 and shCTRL cells, as measured by qPCR. Bars represent the mean with standard deviation (n=3). Statistical significance was tested using unpaired T-test. **(D)** Glucose-stimulated insulin secretion of shHSPA5 and shCTRL cells, depicted as a ratio between high glucose (20 mM) and no glucose exposure. Bars represent mean with standard deviation (n=3). Statistical significance was tested using unpaired T-test. **p≤0.01, ***p≤0.001, ****p≤0.0001.

To evaluate the impact of ER stress on the dialogue between the beta cell and the immune cell compartment, we developed a drug-free model for beta cell ER stress by stable expression of a shRNA specifically targeting the *HSPA5* gene encoding the ER chaperone BiP into EndoC-βH1. As expected, shHSPA5 led to reduced expression of *HSPA5* and to activation of the UPR, as demonstrated by the increased expression of *XBP1s* and *CHOP* (**Figure 1A**). Importantly, despite the increased expression of *CHOP*, no significant difference in the viability was observed between shHSPA5 EndoC-βH1 cells and cells expressing a non-targeting shRNA (shCTRL), as determined by staining for early and late apoptotic cells using Annexin V and 7AAD (**Figure 1B**). However, we observed that *HSPA5* knockdown was accompanied by a decreased insulin gene expression and a reduced capacity to secrete insulin upon glucose stimulation. (**Figures 1C, D**).

**Figure 2:**
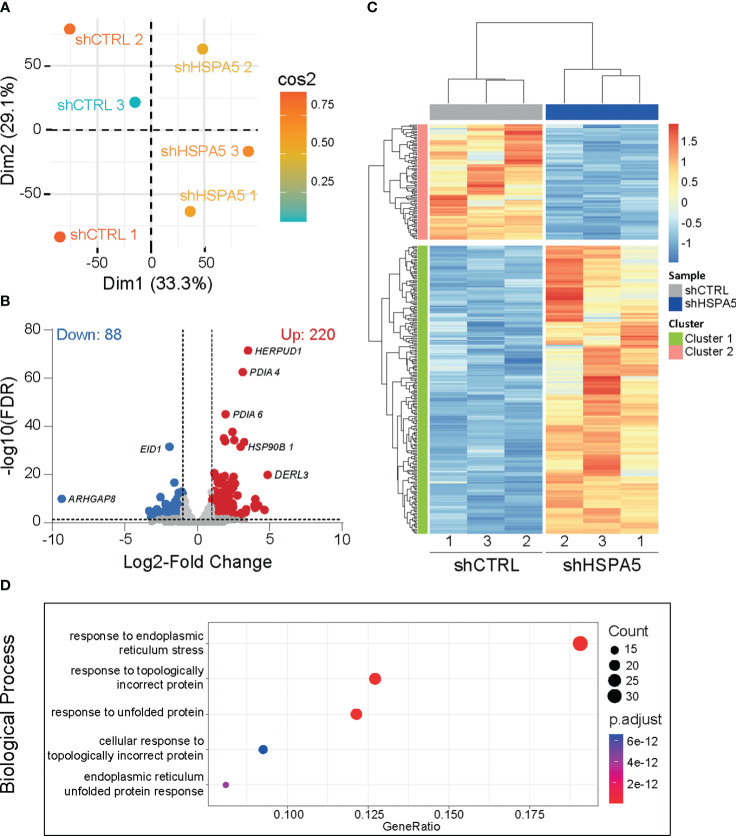
Transcriptomic analysis of shHSPA5 EndoC- βH1 cells. **(A)** PCA plot showing the clustering of shCTRL and shHSPA5 samples based on RNAseq data. **(B)** Volcano plot of differentially expressed genes (threshold of |log2(FC)|≥1.0;FDR ≤ 0.01) in shHSPA5 cells compared to shCTRL cells. Log_2_Fold change values indicate the expression as compared to the shCTRL group. **(C)** Hierarchical clustering of the differentially expressed genes between shCTRL and shHSPA5 cells. **(D)** Gene ontology analysis of the significantly upregulated genes in shHSPA5 cells compared to shCTRL cells, using database GO-BP. Depicted are the top 5 most enriched pathways. The panel on the right indicates the number of genes mapped to the pathway and the adjusted p values from over-representation analysis.

Differential gene expression analysis performed on high-depth RNA-seq data, comparing shHSPA5 EndoC-βH1 cells with shCTRL cells (**Figure 2A**), identified 308 differentially expressed genes, of which 220 were upregulated and 88 were downregulated upon *HSPA5* knockdown (**Figures 2B, C**). Gene ontology analysis using the Gene Ontology knowledgebase GO-BP, (**Figure 2D**), GO-CC, GO-MF and Reactome (**Supplementary Figure 1A**) performed on significantly upregulated genes confirmed enrichment for the ER stress pathway, in line with the qPCR results for *CHOP* and *XBP1s*, as well as enrichment of processes linked to protein synthesis, folding, glycosylation and degradation. The pathways affected by the significantly downregulated genes in shHSPA5 cells were synapse organization and postsynapse organization (**Supplementary Figure 1B**). More specifically, the RNAseq data revealed that *HSPA5* knockdown correlated with an increase in the expression of *HSP90B1*, which encodes GRP94, and as anticipated, a decrease in *INS* expression (**Supplementary Figure 2**). While initially connected to the UPR as a chaperone involved in the processing and transport of secretory proteins, GRP94 has also been described as a component of EVs (39, 40). In addition, we found an upregulation of a subset of genes associated with exosome biogenesis and secretion (**Table 3**), suggesting that ER stress could promote EV secretion in beta cells.

**Table 3:** Genes involved in Reactome pathways related to vesicle formation and release, which are significantly upregulated in shHSPA5 EndoC-βH1 cells.

ESCRT R-HSA-917729	Log_2_FC	FDR
UBAP1	0.522973393	0.008246871
UEVLD	0.821392222	0.002096576
STAM2	0.740560844	0.004729879

Rab regulation of trafficking R-HSA-9007101	Log_2_FC	FDR
DENND2B	0.733235967	0.007189376
DENND5B	0.557016754	0.003159102
RAB1A	0.644423065	0.000297682
RAB5B	0.668093919	0.005343105

### Extracellular vesicles from stressed beta cells contain differentially expressed miRNAs

**Figure 3:**
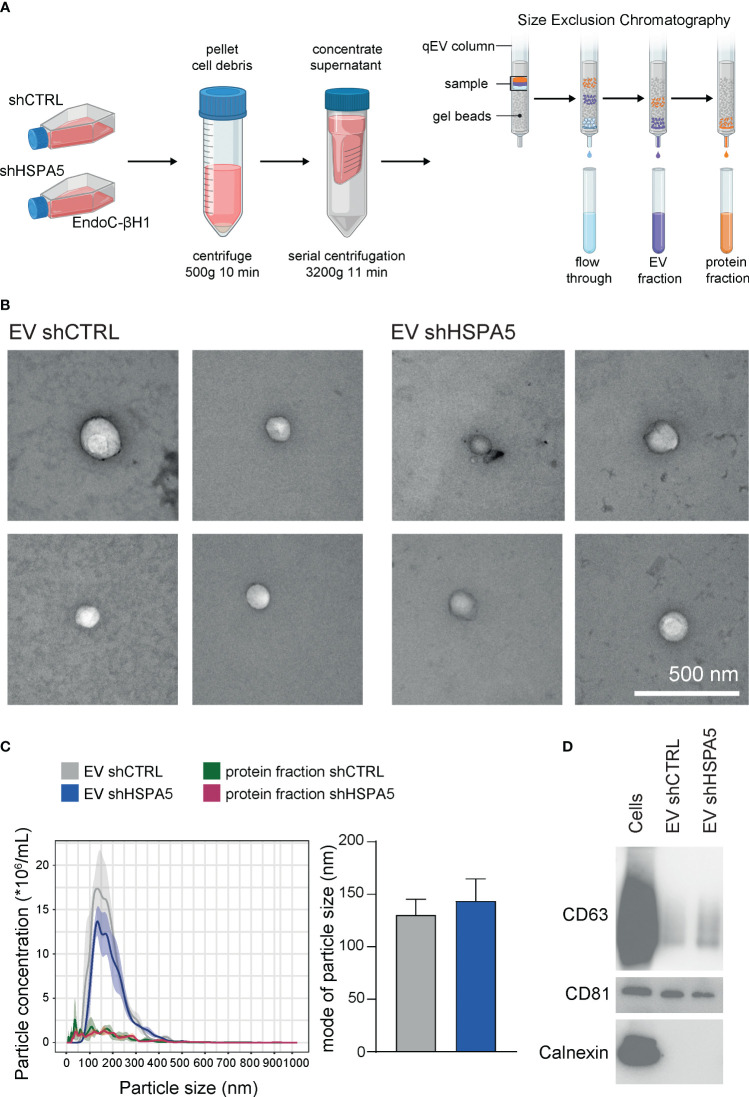
Characterization of EVs isolated from shCTRL and shHSPA5 EndoC-βH1 cells. **(A)** EV isolation workflow. Figure has been created with Biorender.com **(B)** Transmission electron microscopy images of shCTRL- and shHSPA5-derived EVs. The scale bar indicates 500 nm. **(C)** Left: NTA showing the size distribution profile and particle concentration of the EV fractions and protein fractions derived after SEC. The line represents the mean, the ribbon represents the standard error (n=3). Right: Mode of particle size in the EV fractions (n=3). Bars represent the mean with standard deviation. Statistical significance was tested using unpaired T-tests. **(D)** Western blots of CD63 (30-60 kDa), CD81 (20 kDa) and Calnexin (90 kDa) in EndoC-βH1 cell and EV lysates.

To investigate the stressed beta cell vesiculome, EVs were isolated from concentrated conditioned medium of shHSPA5 EndoC-βH1 cells by SEC (**Figure 3A**). Structural characterization of the EV fraction by transmission electron microscopy demonstrated the presence of membrane particles in the range of 30-1000 nm (**Figure 3B**). Nanoparticle tracking analysis showed a size distribution ranging from 100-300 nm in the EV fraction isolated from both shHSPA5 and shCTRL cells, while particles of this size range were depleted in the protein fraction (**Figure 3C**). Quantification by BCA and NTA showed no significant difference in protein content or particle concentration between shHSPA5 EVs and shCTRL EVs (**Supplementary Figure 3**). Moreover, Western blot analyses demonstrated the presence of classical EV membrane-bound markers in the EV lysate, including tetraspanins CD63 and CD81 and absence of ER protein calnexin (**Figure 3D**, **Supplementary Materials**).

**Figure 4:**
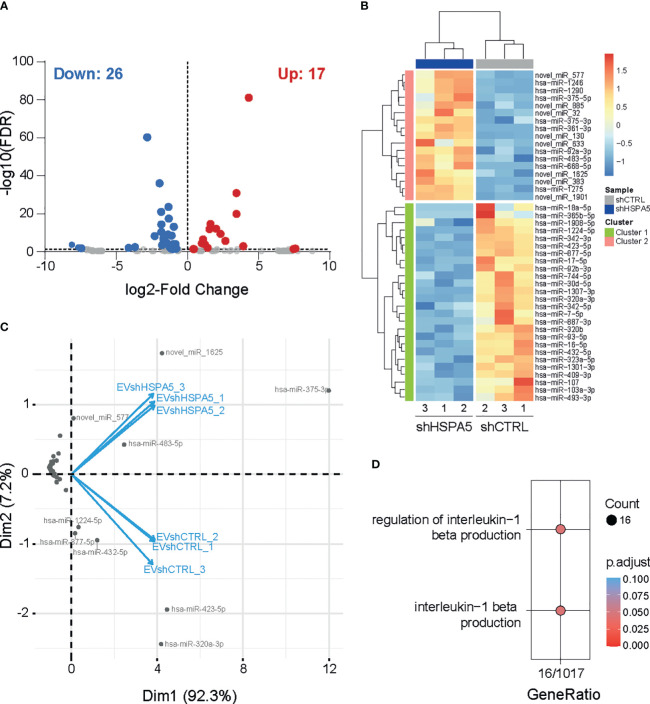
miRNA profiling of EVs from shHSPA5 EndoC-βH1 cells. **(A)** Volcano plot representing significantly up- and down-regulated miRNAs (threshold of FDR ≤ 0.05) in EVs derived from shHSPA5 EndoC-βH1 cells compared to shCTRL EndoC-βH1 cells. Log_2_Fold change values indicate the expression as compared to the shCTRL group. **(B)** Hierarchical clustering of differentially expressed miRNAs between EVs from shCTRL and shHSPA5 cells. **(C)** PCA-biplot (PCA1 and PCA2) of samples and differentially expressed miRNAs in shHSPA5 EVs compared to shCTRL EVs. **(D)** Gene ontology analysis of gene targets of miRNAs under-represented in shHSPA5 EVs revealed involvement in the regulation of IL-1β signaling.

To investigate the EV miRNA cargo, RNA was isolated from shHSPA5 and shCTRL EVs (n=3) and subjected to small RNA sequencing. Following differential miRNA expression analysis, we identified 17 upregulated and 26 downregulated miRNAs in EVs isolated from shHSPA5 cells compared to shCTRL cells (**Figures 4A, B**). Using PCA, a cluster of four miRNAs was identified to be overrepresented in EVs isolated from shHSPA5 cells (**Figure 4C**; **Supplementary Figure 4A**). This cluster consisted of two novel miRNAs (novel_miR-577 and novel_miR-1625) and two known miRNAs (miR-375 and miR-483-5p). miR-375 has previously been associated with beta cell loss in clinical islet transplantation and in streptozotocin-induced and NOD mouse models for diabetes (41, 42), while miR-483-5p originates from the INS-IGF2 locus on human chromosome 11 and has been shown to be upregulated in high glucose and associated with type 2 diabetes (43). Moreover, it has been shown to play a role in maintaining beta cell function and identity through promoting the production of insulin and inhibiting beta cell dedifferentiation (44). In addition, we identified a cluster of five miRNAs, including miR-1224-5p, miR-877-5p, miR-432-5p, miR-423-5p and miR-320a-3p underrepresented in EVs isolated from stressed cells (**Supplementary Figure 4B**). The comparison of the miRNAs identified from secreted EVs with published datasets on human islets (45–47) and primary human beta cells (45, 48) shows that, despite a large heterogeneity illustrating differences in starting material, a large amount were previously identified in primary cells (**Supplementary Figures 5A, B**). Of note, the miRNAs in the PCA clusters mentioned above (miR-375, miR-483-5p, miR-1224-5p, miR-877-5p, miR-432-5p, miR-423-5p and miR-320a) have been described in at least one of these studies (**Supplementary Figure 5C**).

To evaluate the possible biological consequences of EV-containing miRNA secreted by stressed beta cells on neighboring immune cells, we investigated the targets of these miRNAs. *In silico* analysis using miRWalk revealed four potential gene targets of miR-375 and/or miR-483-5p, and 1103 genes targeted by miR-1224-5p, miR-877-5p, miR-432-5p, miR-423-5p and/or miR-320a-3p. To dissect the molecular mechanisms regulated by the identified miRNAs, we performed gene ontology analysis on experimentally validated mRNA targets of miRNAs that were differentially upregulated or downregulated in EVs from shHSPA5 cells, compared to EVs derived from shCTRL cells (online **Supplementary Information** https://figshare.com/s/54809507652156a18c90). Interestingly, among the various pathways potentially affected, we observed that miRNAs downregulated in EVs from shHSPA5 cells are involved in IL-1β signaling (**Figure 4D**), suggesting a potential role of EV miRNA in the crosstalk between stressed beta cells and the innate immune cell compartment.

### Extracellular vesicles from stressed beta cells promote activation of monocytes

**Figure 5:**
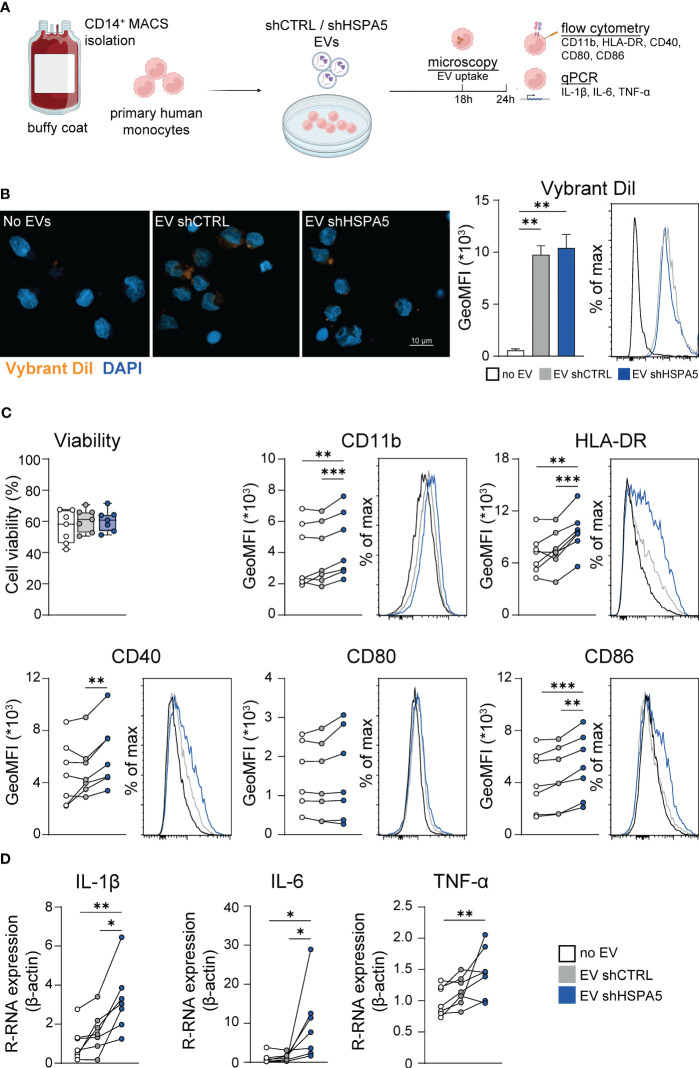
EVs from stressed EndoC-βH1 cells are taken up by and promote the activation of monocytes. **(A)** Workflow displaying the treatment of primary human monocytes with EVs derived from shCTRL and shHSPA5 EndoC-βH1 cells. Figure has been created with Biorender.com **(B)** Left: Confocal imaging demonstrating the uptake of Vybrant DiI-stained EVs by monocytes after 18h. Nuclei were stained with DAPI. The scale bar indicates 10 µm. Right: Quantification of monocyte uptake of EVs from shCTRL (grey) or shHSPA5 (blue) EndoC-βH1 cells measured by flow cytometry. Bars represent the mean with standard deviation (n=2). Statistical significance was tested using one way ANOVA followed by Tukey’s multiple comparisons test. **(C)** Viability of monocytes, determined by Zombie-NIR staining. Each dot represents one donor. Data are represented as boxplots with median and whiskers from min to max. Cell surface expression of CD11b, HLA-DR, CD40, CD80 and CD86 on monocytes treated with no EVs (white), EVs from shCTRL (grey) or EVs from shHSPA5 (blue) EndoC-βH1 cells measured by flow cytometry. Each line represents the measurement from one monocyte donor. Significance was tested using a one way ANOVA followed by Tukey’s multiple comparisons test. **(D)** Gene expression of IL-1β, IL-6 and TNF-α in monocytes incubated with no EVs (white), EVs from shCTRL (grey) or EVs from shHSPA5 (blue) EndoC-βH1 cells as measured by qPCR, shown as relative mRNA expression normalized to β-actin expression. Each line represents one monocyte donor. Significance was tested using a one way ANOVA followed by Tukey’s multiple comparisons test. *p≤0.05, **p≤0.01, ***p≤0.001.

Assuming that innate immune cells are the first immune cells recruited to stressed cells, we investigated the effect of EVs released from stressed beta cells on primary human monocytes. The knockdown efficiency and ER stress levels in the EndoC-βH1 cells that were used to produce the different EV batches were verified (**Supplementary Figures 6A, B**). Monocytes isolated from blood of healthy donors were exposed to EVs isolated from shCTRL and shHSPA5 EndoC-βH1 cells (**Figure 5A**) and EV uptake by monocytes was confirmed by confocal microscopy after overnight incubation with Vybrant DiI-labelled vesicles (**Figure 5B**). Quantification by flow cytometry showed no statistically significant difference in uptake between EVs from shCTRL and shHSPA5 cells. Next, monocyte activation was assessed by flow cytometry and qPCR. Monocytes treated with EVs from shHSPA5 cells significantly increased surface expression of integrin CD11b, HLA-DR, and costimulatory molecules CD40 and CD86, as compared to monocytes treated with EVs from shCTRL cells (**Figure 5C**). These results suggest that EVs from stressed cells can promote monocyte capacity to adhere, invade, present antigens and activate adaptive immune cells. Furthermore, qPCR results showed that monocytes treated with shHSPA5 expressing EndoC-βH1-derived EVs significantly increased mRNA levels of IL-1β and IL-6, and there was a tendency towards elevated TNF-α levels (**Figure 5D**). Increased activation of monocytes was consistently observed between different donors and batches of EVs (**Supplementary Figures 6C, D**). Altogether, these findings suggest that EVs from stressed beta cells can steer monocyte activation, promoting a more proinflammatory phenotype.

## Discussion

*In vitro* models of ER stress typically rely on the use of chemical inducers, such as thapsigargin or tunicamycin, or cytokine cocktails. While these approaches have provided valuable insights, they hamper the examination of intercellular communication. The model developed in this study, based on modulating *HSPA5* expression, allows for i) examination of ER stress without additional undesired effects of ER calcium depletion or inflammation and ii) investigation of the effect of cellular stress on neighboring cells while avoiding drug carry-over effects on recipient cells.

Cellular stress activates various coping mechanisms, including increased formation of EVs (49–51). The generation of EVs is dependent on the endosomal sorting complex required for transport (ESCRT) or sphingomyelinases, while their secretion is facilitated by Rab GTPases, Ral GTPases, SNARE proteins and V-type ATPase (18, 52). Previous studies have shown that the IRE1α and PERK pathway of the ER stress response can promote the formation of multivesicular bodies and upregulate the expression of sphingomyelinase SMPD3 (16, 50). In this study, ER stress induced by *HSPA5* knockdown resulted in increased expression of genes involved in the ESCRT complex (UBAP1, UEVLD and STAM2) and in transport of vesicles to the plasma membrane (DENND2B, DENND5B, RAB1A and RAB5B). 

The release of EVs during ER stress may serve as a way to dispose of misfolded or erroneous proteins, a phenomenon that has been demonstrated in protein aggregate-associated neurodegenerative diseases (53, 54). The identification of various DAMPs, including HMBG1, heat shock proteins and ceramides inside EVs from stressed cells indicate that the release of EVs may also be a means to alert the immune system (49, 51, 55). Recent studies in the cancer field emphasize the immunomodulatory capacities of EVs, showing that EVs released from cancer cells under immunogenic stress are able to shape the immune response in the tumor microenvironment (56–58). Here, we demonstrate that EVs from stressed beta cells can induce a proinflammatory response in monocytes, indicating that the beta cells could actually be the spark initiating the development of autoimmunity by activating innate immune cells.

Recently, increasing evidence suggests that EVs play an important role in the communication between beta cells and the immune system, as reviewed by Grieco GE et al. (9). Moreover, previous studies have identified the presence of cytokines, chemokines, miRNAs, immunostimulatory chaperones and T1D autoantigens as part of the beta cell or islet EV cargo (19, 46, 59–65). However, the composition of beta cell-derived EVs has only been studied in conditions of cytokine treatment, UV exposure and hypoxia, and not specifically for ER stress (59). In the present study, we found that ER stress changes the cargo of beta cell EVs, as demonstrated by the identification of 43 differentially expressed miRNAs. In addition to enrichment of miRNAs associated with beta cell loss, confirming our model for beta cell stress, we found that EVs from stressed beta cells contain a reduced abundance of IL-1β signaling regulating miRNAs. The latter may suggest that, in healthy conditions, beta cells actively maintain an immune tolerant environment, whereas, in this model, ER stress seems to release the handbrake that prevents monocyte activation. Of note, the observed effects might also be attributed to the increased expression of miR-375, as it has been shown to promote the production of IL-1β, IL-6 and TNF-α in acinar cells and macrophages (66, 67), as well as the other EV components (non-coding RNA, proteins, lipids).

The effect of beta cell EVs on innate immune cells has previously been studied in splenocytes or bone marrow-derived DCs from NOD mice, which were stimulated with EVs isolated from MIN6 cells or rat islets (60, 68), and primary human monocytes treated with EVs isolated from human pancreatic islets (69). In line with our results, these studies reported similar phenotypic changes, including increases in HLA class II, CD40, TNF-α, IL-1β, IL-6 and IL-10. Moreover, EVs isolated from cytokine-stimulated beta cells or islets have been shown to increase the expression of inflammatory markers on recipient NOD mouse-derived dendritic cells (DCs) (59, 60). Although the increase in cytokine production was substantially higher than reported in this study, it cannot be ruled out that some of these effects, besides the use of different target cells, may have been due to cytokine carry-over, since cytokines from the stimulation could have been encapsulated into the EVs (70).

More than just participating to the proinflammatory milieu, the upregulation of antigen-presenting -related factors (HLA-DR, CD40 and CD86) observed on the recipient cells suggests a much broader effect and major consequences in the cascade of events leading to the T cell-mediated destruction of beta cells. Upregulation of these cell surface markers in monocytes has been associated with an increased frequency of memory T cells and the development of recent-onset T1D in children (71). In transplantation, a similar change in phenotype, induced by vesicles from the graft donor DCs, was shown to be critical in alloreactive T cell activation and graft rejection (72). Although these studies illustrate a functional role of monocytes in T cell activation, further studies are needed to investigate the impact of EVs from stressed beta cells, via monocytes or other antigen-presenting cells, on T cell subsets in more detail.

Altogether, these data highlight the importance of the beta cell vesicle content in the development of islet inflammation and illustrate the need to devise novel therapeutic strategies to alleviate cellular stress, prevent beta cell dysfunction and destruction.

## Data availability statement

The datasets generated for this study can be found in figshare: https://figshare.com/s/54809507652156a18c90.

## Ethics statement

Ethical approval was not required for the studies on humans in7nbsp;accordance with the local legislation and institutional requirements because only commercially available established cell lines were used.

## Author contributions

MD: Writing – original draft, Data curation, Investigation, Methodology, Writing – review & editing. JL: Writing – original draft, Data curation, Investigation, Methodology, Writing – review & editing. XP: Writing – original draft, Data curation, Investigation, Methodology, Writing – review & editing. RF: Writing – original draft, Data curation, Formal analysis, Software, Writing – review & editing. AE-M: Writing – original draft, Investigation, Methodology, Writing – review & editing. KK: Writing – review & editing, Investigation, Methodology, Writing – original draft. BGi: Writing – review & editing, Investigation, Methodology, Writing – original draft. BGu: Writing – original draft, Writing – review & editing, Methodology, Resources, Supervision. AZ: Writing – original draft, Writing – review & editing, Funding acquisition, Methodology, Resources, Supervision.
