# Supplementary material for: Extracellular vesicles derived from stressed beta cells mediate monocyte activation and contribute to islet inflammation
Source: Front Immunol. 2024 Jul 24;15:1393248. doi: 10.3389/fimmu.2024.1393248 (PMC11303142; doi:10.3389/fimmu.2024.1393248)
Supplement: Supplementary file 1 [file DataSheet_1.pdf]

Supplementary Material

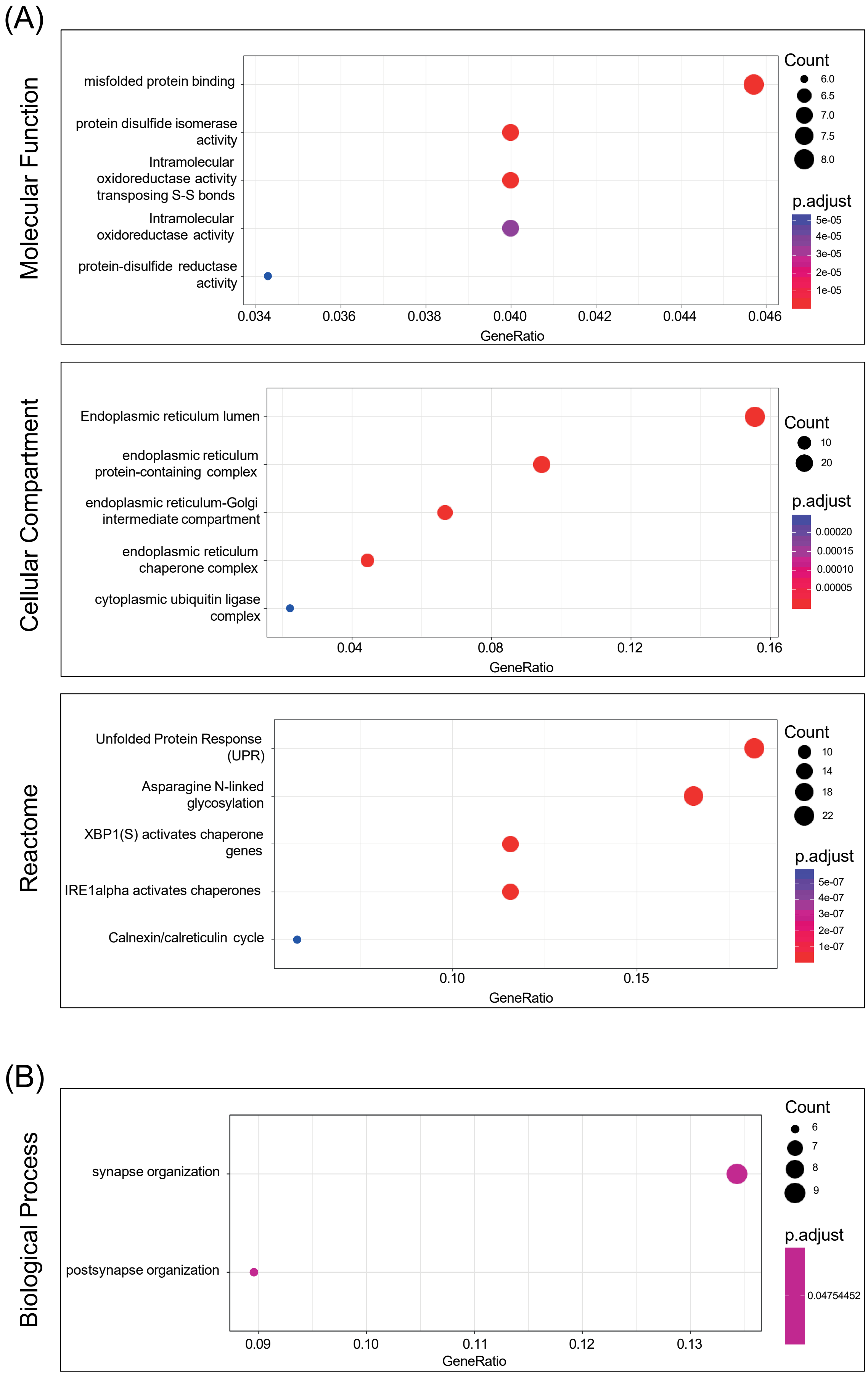
(A)
Molecular Function
misfolded protein binding
protein disulfide isomerase activity
Intramolecular oxidoreductase activity transposing S-S bonds
Intramolecular oxidoreductase activity
protein-disulfide reductase activity
0.034
0.036
0.038
0.040
0.042
0.044
0.046
GeneRatio
Count
6.0
6.5
7.0
7.5
8.0
p.adjust
5e-05
4e-05
3e-05
2e-05
1e-05
Cellular Compartment
Endoplasmic reticulum lumen
endoplasmic reticulum protein-containing complex
endoplasmic reticulum-Golgi intermediate compartment
endoplasmic reticulum chaperone complex
cytoplasmic ubiquitin ligase complex
0.04
0.08
0.12
0.16
GeneRatio
Count
10
20
p.adjust
0.00020
0.00015
0.00010
0.00005
Reactome
Unfolded Protein Response (UPR)
Asparagine N-linked glycosylation
XBP1(S) activates chaperone genes
IRE1alpha activates chaperones
Calnexin/calreticulin cycle
0.10
0.15
GeneRatio
Count
10
14
18
22
p.adjust
5e-07
4e-07
3e-07
2e-07
1e-07
(B)
Biological Process
synapse organization
postsynapse organization
0.09
0.10
0.11
0.12
0.13
GeneRatio
Count
6
7
8
9
p.adjust
0.04754452

Supplementary Figure 1: Gene ontology analysis of differentially expressed genes between shHSPA5 and shCTRL EndoC-βH1 cells. **(A)** Gene ontology analysis of the list of genes significantly upregulated in shHSPA5 cells, compared to shCTRL cells, using GO-MF, GO-CC and Reactome. Depicted are the top 5 most enriched pathways. The panel on the right indicates the number of genes mapped to the pathway and the adjusted p values from over-representation analysis. **(B)** Gene ontology analysis of the list of genes significantly downregulated in shHSPA5 cells, compared to shCTRL cells, using GO-BP, GO-MF, GO-CC and Reactome. Only two pathways in GO-BP were significantly affected (adjusted p value ≤ 0.05).

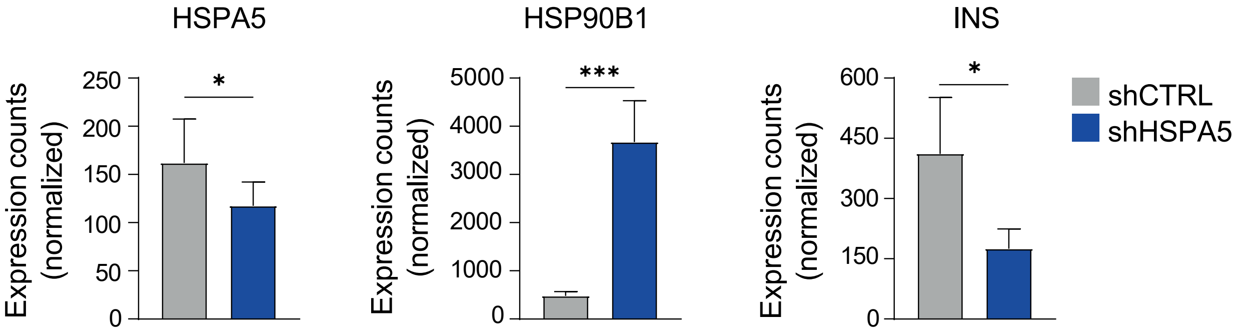

Supplementary Figure 2: Gene expression of HSPA5, HSP90B1 and INS in shHSPA5 and shCTRL EndoC-βH1 cells. Expression levels are shown as normalized counts (FPKM) from RNA sequencing. Bars represent the mean with standard deviation (n=3). Statistical significance was tested using ratio paired T-tests.

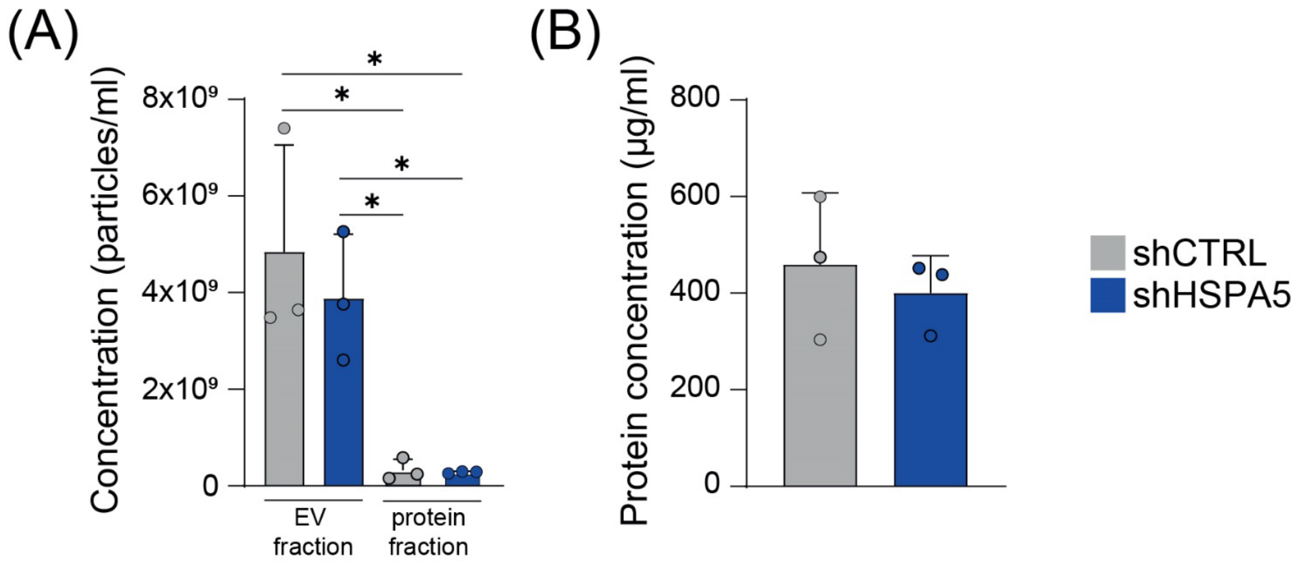

Supplementary Figure 3: Quantification of EVs by NTA and BCA
(A) Number of particles/ml as measured by NTA. Bars represent mean with standard deviation (n=3). Statistical significance was tested using one-way ANOVA. **(B)** Protein concentration of lyzed EVs measured by BCA. Bars represent mean with standard deviation (n=3). Statistical significance was tested using unpaired T-test.

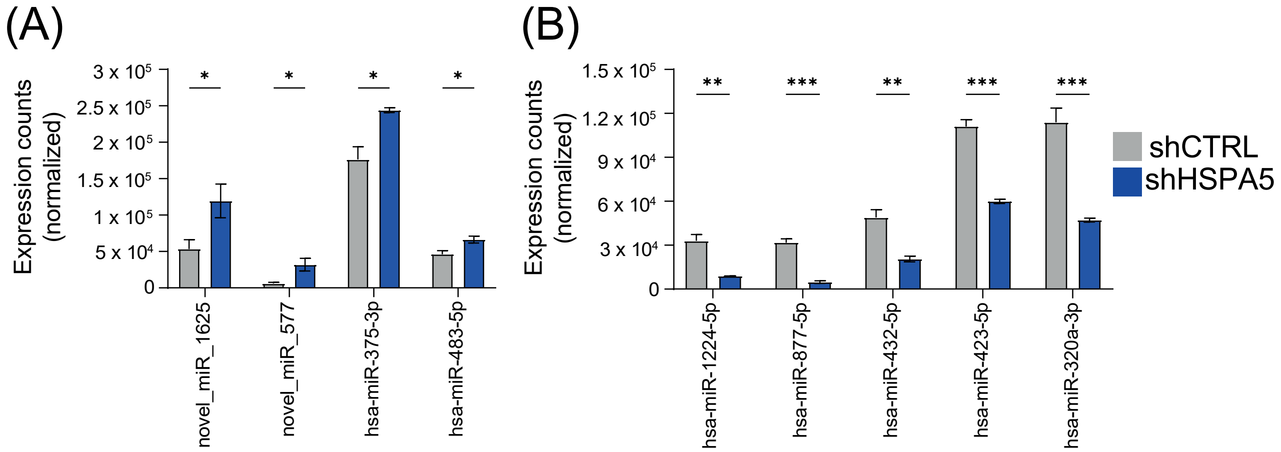

Supplementary Figure 4: Expression levels of miRNAs differentially expressed between EVs from shHSPA5 EndoC-βH1 cells and shCTRL cells, shown in PCA-biplot. Expression levels are shown as normalized counts from small RNA sequencing. **(A)** Normalized counts for Dim1 positive/Dim2 negative miRNAs from PCA-biplot (see Figure 3C) in EVs derived from shHSPA5 EndoC-βH1 cells and shCTRL cells. Bars represent the mean with standard deviation (n=3). Statistical significance was tested using unpaired T-tests. **(B)** Normalized counts for Dim1 positive/Dim2 positive miRNAs from PCA-biplot (see Figure 3C) in EVs derived from shHSPA5 EndoC-βH1 cells and shCTRL cells. Bars represent the mean with standard deviation (n=3). Statistical significance was tested using unpaired T-tests.

(A)

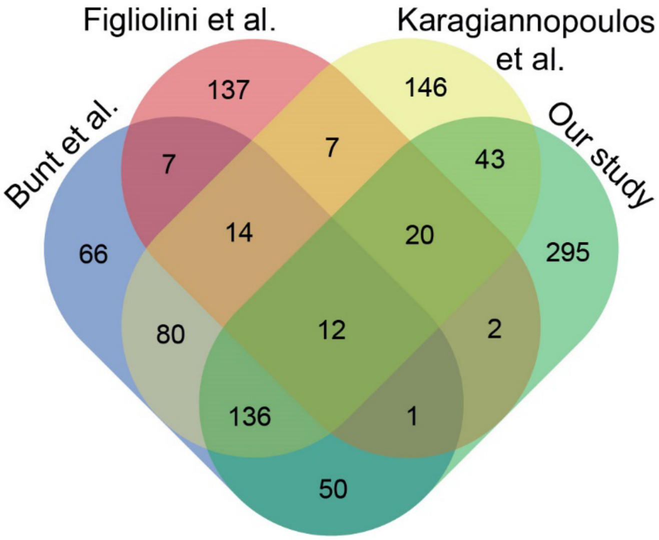

(B)

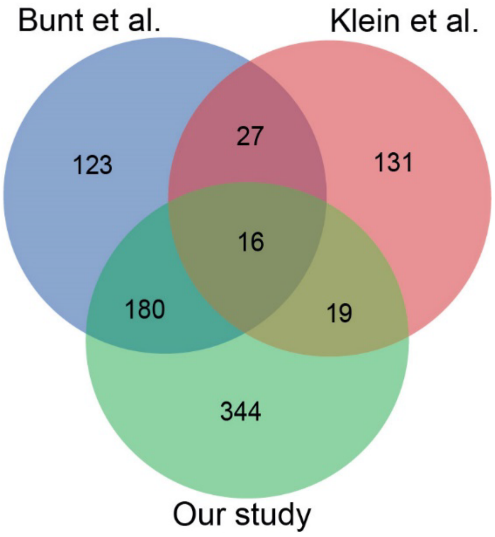

(C)

miRNAs highlighted in PCA plot:	Overlap with primary islets studies:	Overlap with primary beta cells studies:
miR-375	in all studies (1-3)	in all studies (1, 4)
miR-483-5p	Karagiannopoulos et al. (3), Figliolini et al. (2)	Klein et al. (4)
miR-1224-5p	Bunt et al. (1), Karagiannopoulos et al. (3)	-
miR-877-5p	Karagiannopoulos et al. (3)	-
miR-432-5p	Bunt et al. (1), Karagiannopoulos et al. (3)	Bunt et al. (1)
miR-423-5p	in all studies (1-3)	In all studies (1, 4)
miR-320a	Bunt et al. (1), Karagiannopoulos et al. (3)	Bunt et al. (1)

Supplementary Figure 5: Comparison of miRNAs found in EVs from shBiP and shCTRL EndoC-βH1 cells with published miRNA datasets on primary human islets and beta cells. (**A**) Overlap between miRNAs detected in our study versus studies with primary human islets (**B**) and primary beta cells. (**C**) Validation of the clusters of miRNAs identified in PCA (**Figure 4C**) in datasets on primary human cells.

(A)

EV batch	HSPA5 knockdown	ER stress (XBP1s)	Monocyte activation
1	27.8%	32.1 fold upregulation	+
2	41.7%	40.1 fold upregulation	+

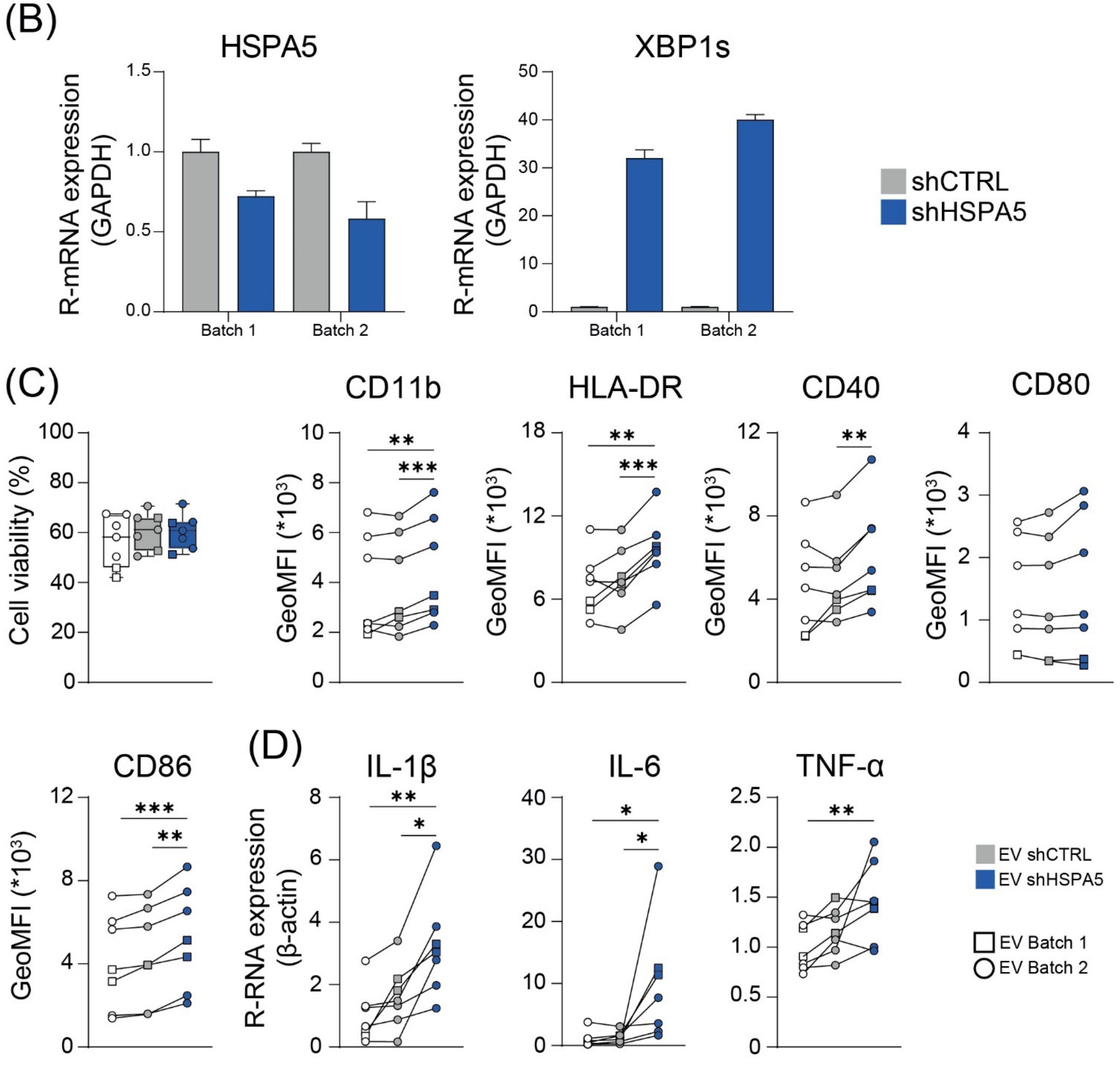

Supplementary Figure 6: Overview of EV characteristics and monocyte responses using different EV batches isolated from shHSPA5 and shCTRL EndoC-βH1 cells. **(A)** Knockdown efficiency and upregulation of *XBP1s* in cells used for the production of EVs for monocyte stimulation experiments. **(B)** *HSPA5* and *XBP1s* expression in shCTRL and shHSPA5 EndoC-βH1 cells used for production of EV batch 1 and 2, as evaluated by qPCR. Bars represent the mean with standard deviation (n=3, technical replicates). **(C)** Viability of monocytes treated with EVs from batch 1 (squares) or batch 2 (dots), determined by Zombie-NIR staining. Each dot represents one donor. Data are represented as boxplots with median and whiskers from min to max. Cell surface expression of CD11b, HLA-DR, CD40, CD80 and CD86 on monocytes treated with no EVs (white), EVs from batch 1 (squares) or batch 2 (dots) from shCTRL (grey) or shHSPA5 (blue) EndoC-βH1 cells measured by flow cytometry. Each line represents the measurement from one monocyte donor. Significance was tested using a one way ANOVA followed by Tukey's multiple comparisons test. **(D)** Gene expression of IL-1β, IL-6 and TNF-α in monocytes incubated with no EVs (white), EVs from batch 1 (squares) or batch 2 (dots) from shCTRL (grey) or shHSPA5 (blue) EndoC-βH1 cells as measured by qPCR, shown as relative mRNA expression normalized to β-actin expression. Each line represents one monocyte donor. Significance was tested using a one way ANOVA followed by Tukey's multiple comparisons test.

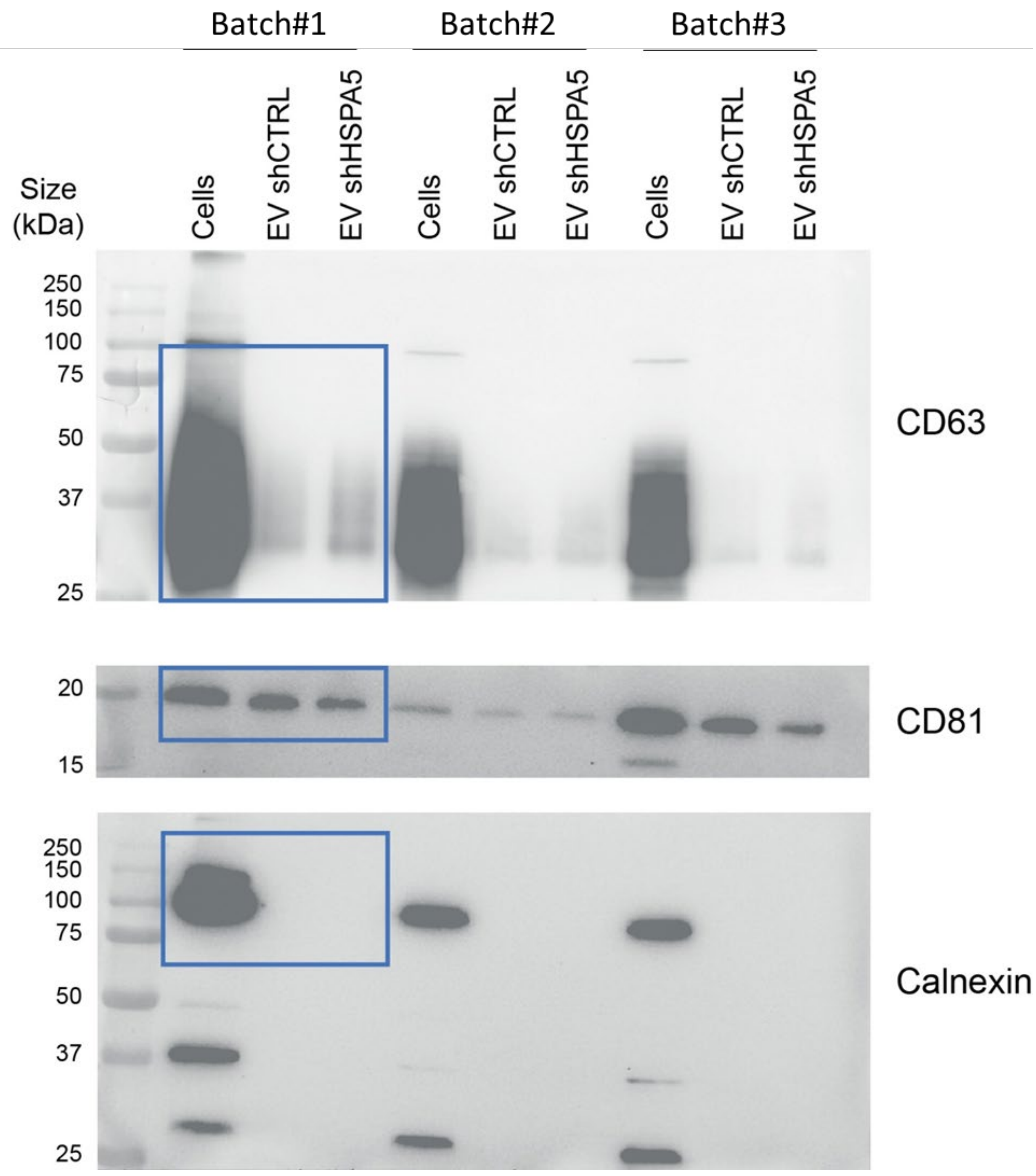

Supplementary materials: Full Western blots of CD63 (30-60 kDa), CD81 (20 kDa) and calnexin (90 kDa) in EndoC- βH1 cell lysates and EV lysates.

References Supplementary Material

1. van de Bunt M., Gaulton K.J., Parts L., Moran I., Johnson P.R., Lindgren C.M., et al. The miRNA Profile of Human Pancreatic Islets and Beta-Cells and Relationship to Type 2 Diabetes Pathogenesis. PloS one. 2013;8(1):e55272. doi: 10.1371/journal.pone.0055272

2. Figliolini F., Cantaluppi V., De Lena M., Beltramo S., Romagnoli R., Salizzoni M., et al. Isolation, Characterization and Potential Role in Beta Cell-Endothelium Cross-Talk of Extracellular Vesicles Released from Human Pancreatic Islets. PloS one. 2014;9(7):e102521. doi: 10.1371/journal.pone.0102521

3. Karagiannopoulos A., Esguerra J.L.S., Pedersen M.G., Wendt A., Prasad R.B., Eliasson L. Human pancreatic islet miRNA-mRNA networks of altered miRNAs due to glycemic status. iScience. 2022;25(4):103995. doi: 10.1016/j.isci.2022.103995

4. Klein D., Misawa R., Bravo-Egana V., Vargas N., Rosero S., Piroso J., et al. MicroRNA Expression in Alpha and Beta Cells of Human Pancreatic Islets. PloS one. 2013;8(1):e55064. doi: 10.1371/journal.pone.0055064
